# Clinical characteristics and pregnancy outcomes of women diagnosed with SARS-CoV-2 in London’s most ethnically diverse borough: A cross-sectional study

**DOI:** 10.1177/1753495X20985403

**Published:** 2021-01-21

**Authors:** Jack Milln, Samuel Heard, Kirun Gunganah, Luxmimalar Velauthar, Ferha Saeed

**Affiliations:** 1 Department of Endocrinology and Diabetes, Queen Mary University of London, London, UK; 2 Department of Endocrinology and Diabetes, Newham University Hospital, London; 3 Department of Obstetrics and Gynaecology, Newham University Hospital, London

**Keywords:** Coronavirus, SARS-CoV-2, COVID-19, pregnancy, ethnicity, birthweight

## Abstract

**Introduction:**

It is unclear whether pregnant women from ethnic minority groups and with metabolic disorders are disproportionately affected by SARS-CoV-2 infection within deprived areas. No previous studies have compared pregnancy outcomes with an appropriate comparator group.

**Methods:**

Cross-sectional study of 32 women with SARS-CoV-2 compared to background departmental figures from the three months prior to the outbreak. Clinical characteristics were compared to the UK Obstetric Surveillance System report.

**Results:**

Estimated incidence was 10 times the national average (50.3 vs 4.9 per 1000 maternities). Women from Black (OR, 95% CI: 3.01, 1.08–7.38) and Asian (OR, 95% CI: 2.68, 1.23–6.05) ethnic groups were over-represented; however, there was no association with metabolic disorders. Babies born to women diagnosed with coronavirus were more likely to be born premature, or by caesarean delivery, however there was no difference in birthweight centile for gestational age.

**Conclusion:**

Women from Black and Asian backgrounds are disproportionately affected, even within an area of high ethnic diversity. Mothers do not appear more severely affected than women nationally; however, babies are more likely to be born preterm, or by caesarean delivery, compared to usual departmental figures. It is unclear whether this is due to increased intervention or a direct result of coronavirus infection.

## Introduction

There is a current pandemic of novel coronavirus disease (SARS-CoV-2, or COVID-19). Previous pandemics of similar pathogens such as SARS-CoV-1, Middle Eastern Respiratory Syndrome (MERS-CoV) and influenza A/H1N1 have caused greater illness severity in pregnant women and been associated with adverse pregnancy outcomes.^1–4^ The recent report from the national UK Obstetric Surveillance System (UKOSS)^5^ does not suggest a similar pattern of morbidity and mortality for this novel coronavirus in pregnancy, in line with other recent international reports.^6^ Whilst these reports are broadly reassuring, none have included a direct comparator group to assess pregnancy outcomes. Therefore, the impact of SARS-CoV-2 infection of pregnancy outcomes is still not known. The UKOSS study also highlighted an association between risk of hospitalisation with coronavirus infection and both ethnicity and underlying conditions such as obesity and diabetes. This supports other reports that these groups are more likely to develop severe infection and die from COVID-19 infection.^7–10^ However, it is not fully understood whether these characteristics independently confer risk or whether the association is found because these characteristics are more common in severely affected areas due to wider issues such as deprivation. It is therefore important to explore whether these disparities still exist within areas where these risk factors are particularly prevalent.

Newham, a borough in East London, is home to the UK’s most ethnically diverse population, and reports the highest age-standardised mortality rate from coronavirus disease in the UK at 144.3 per 100,000 population.^11^ Overall, 72.9% of the population are from ethnic minority backgrounds, compared with 46.2% across London, and 16.7% across England and Wales.^12,13^ Almost half (47.5%) of the population were born outside of the UK, and only 58.6% consider English their main language. It is one of the most economically deprived boroughs in London, and in the bottom 10% most deprived boroughs in the country.^12^ The prevalence of type 2 diabetes is 8.6%, more than double that in more affluent boroughs in West London.^14^ The maternity unit at Newham University Hospital has a robust system of collecting baseline pregnancy outcomes data and demographic information. This presents a unique opportunity to explore whether pregnancy outcomes and risk factors in a national survey are reflected on the local level in a high-risk area.

The aim of this study, therefore, was to describe the characteristics and pregnancy outcomes of women living in a borough with a high proportion of underlying risk factors. These were compared to local baseline departmental figures in the immediate pre-COVID-19 era, and the recent national obstetric surveillance report, to determine whether women from high-risk groups were disproportionately affected.

## Methods

### Setting

Newham University Hospital, a district general hospital, provides secondary level care. The maternity unit is responsible for approximately 5,600 deliveries per year.

### Study design

This is a retrospective cross-sectional study of all pregnant women diagnosed with SARS-CoV-2 and a direct comparator group of women delivering at the same maternity unit in the three months leading up to the UK outbreak. Explicit consent was not sought due to the retrospective nature of the audit and absence of identifying data presented. Data were analysed and interpreted by the authors. Approval was granted by the Barts Health NHS Trust institutional ethic committee (ID: 133659). No funding was sought and the authors declare no conflicts of interest. All cases have been reported by the UKOSS. However, these are reported along with other national data and not seen as a discrete data set.

### Participants

Inclusion criteria: All pregnant women with a clinical or laboratory-confirmed diagnosis of SARS-CoV-2 diagnosed at Newham University Hospital between 12 March 2020 until 22 April 2020.

Comparator group: All deliveries at Newham University Hospital between December 2019 and February 2020 inclusive. Clinical characteristics were compared to the UKOSS report.^5^

### Data collection

We obtained the electronic and paper medical records of participants and compiled the demographic, clinical and outcome data for all pregnant women with a clinical or laboratory-confirmed diagnosis of SARS-CoV-2 during the study period. Universal screening for the inpatient pregnant population was not in place during the study period and women were only tested if they displayed typical symptoms. Laboratory confirmation of SARS-CoV-2 was defined as a positive result on the polymerase chain reaction assay of maternal nasopharyngeal swab. A clinical diagnosis was made by clinicians if the participant fulfilled typical clinical, laboratory or radiological features of the illness, accounting for the false false negative rate rate of the viral nucleic acid test. No protocol was in place for testing neonates for SARS-CoV-19 and this was based on clinical judgement of the neonatal team. Neonates underwent a nasopharyngeal swab with identical laboratory analysis as maternal swabs. This may have been done before or after initiation of breastfeeding.

Demographic data were extracted from the initial electronic antenatal booking information and clinical symptoms/signs from the electronic and paper medical notes recorded during admission. Laboratory data was taken from electronic notes (CRS Cerner) and maternal and neonatal outcome data extracted from the standard departmental electronic records.

Demographic and pregnancy outcome data of women delivering at the same maternity unit during the three months immediately prior to the outbreak (1st December 2019 to 29th February 2020, inclusive) were extracted from the departmental electronic records. Some data from the UKOSS report was used as the comparator group for clinical characteristics.

### Study outcomes

The main outcomes were: incidence of coronavirus infection, proportion of women from ethnic minority groups, proportion of women with obesity and diabetes, admission to critical care, perinatal death, birthweight centile and median gestational age at delivery.

### Sample size and statistical analysis

No formal power calculation was undertaken as the sample size was dictated by the number of cases diagnosed in the study period. Incidence was calculated using the number of cases or coronavirus infection divided by the number of maternities (deliveries) during the study period. Participants’ demographic, clinical characteristics and pregnancy outcomes were summarised as means and standard deviations, or medians and interquartile ranges, for continuous variables. Categorical data were summarised using numbers and proportions. We present crude odds ratios (ORs) between our cohort and our background departmental population, with 95% confidence intervals. We used Student’s *t* test and Pearson’s chi-square (and Fisher’s exact tests) to detect differences. We calculated birthweight centiles using the INTERGROWTH-21 application,^15^ using birthweight, gestational age at delivery, and sex of the baby as variables.

## Results

### Participant characteristics

The characteristics of women diagnosed with SARS-CoV-2 (12 March 2020 to 22 April 2020) and our departmental comparator group are displayed in Table 1. Almost three quarters of women were born outside of the UK and a quarter required an advocate due to poor understanding of English; no comparison could be made with departmental rates due to missing data. There was an association with women from Asian and Black ethnic groups. Notably, all five women whose ethnic group was classed as ‘White’ were from Eastern Europe, all born outside the UK, with two requiring a language advocate. There was no significant difference in maternal age, booking BMI, or proportion with obesity, type 2 or gestational diabetes compared to women delivering in the three months prior to the outbreak.

**Table 1.** Characteristics of pregnant women diagnosed with SARS-CoV-2 and baseline departmental figures from women delivering between December 2019 and February 2020.

	Baseline departmental figuresNumber (%)	SARS-CoV-2 infection	Odds ratio (95% CI)	*p*-value
	*n* = 1284	*n* = 32		
Demographics				
Age (years; mean ±SD)	29.7 (±5.5)	30.1 (±4.5)	–	0.66
Ethnicity				
White	298 (23)	5 (16)	–	
Asian	493(38)	20 (63)	2.67 (1.23–6.05)	<0.01
Black	109 (8.5)	7 (22)	3.02 (1.08–7.38)	<0.01
Chinese/other	319 (25)	0 (0)	–	
Mixed	17 (1.3)	0 (0)	–	
Missing	48 (3.2)	0 (0)	–	
Born in UK – Yes		9 (28)		
Born in UK – No		23 (72)		
Advocate required – Yes		9 (28)		
Advocate required – No		23 (72)		
				
Anthropometrics				
Booking BMI kg/m^2^, mean (±SD)	26.7 (±6.5)	27.5 (±5.9)		0.49
Co-morbidities				
Obesity (BMI ≥30 kg/m^2^)	257 (20)	8 (25)	1.33 (0.51–3.11)	0.49
T2DM or GDM	197 (15)	7 (22)	1.54 (0.56–3.74)	0.31

BMI: body mass index. T2DM; type 2 diabetes mellitus. GDM: gestational diabetes mellitus.

### Clinical characteristics

The clinical characteristics of women diagnosed with SARS-CoV-2 at Newham University Hospital are displayed in Table 2, with comparison figures from the UKOSS report.^5^ There were 32 cases and 636 deliveries during the study period, giving an estimated incidence of 50.3 cases per 1000 maternities. The majority were laboratory-confirmed cases, and two were clinical diagnoses. Of the clinical diagnoses, one woman was self-isolating prior to her deterioration at home, and another had typical symptoms and radiological features during hospital admission. One woman was admitted to critical care; she deteriorated post-operatively following an emergency caesarean delivery at 33 weeks and 6 days of gestation due to maternal hypoxia. She required non-invasive respiratory support and was discharged from the critical care unit after 24 h. Four other women required oxygen therapy.

**Table 2.** Clinical, biochemical, radiological and management characteristics of pregnant women diagnosed with SARS-CoV-2.

	SARS-CoV-2 infection NewhamNumber (%)	UKOSS report
	*n* = 32	*n* = 427
Estimated incidence (per 1000 maternities)	50.3	4.9
Diagnosis with admission for coronavirus symptoms	16 (50)	–
Diagnosis in hospital after admission for obstetric reasons	16 (50)	–
Trimester at diagnosis		
1st or 2nd	3 (9.4)	82 (19)
3rd	27 (84)	312 (73)
Peripartum	2 (6.3)	30 (7.0)
Clinical		
Median (IQR) time between onset of symptoms and diagnosis (days)	1 (0–7)	–
Fever	26 (81)	280 (66)
Cough	17 (53)	244 (57)
Shortness of breath	10 (31)	155 (36)
Lethargy	6 (19)	65 (15)
Limb pain	7 (22)	50 (12)
Sore throat	1 (3.1)	43 (10)
Headache	4 (13)	67 (16)
Biochemical		
SARS-CoV-2 PCR swab positive	30 (94)	
Lymphopenia	7 (22)	
Median (IQR) Lymphocyte count (×10^9^/L)	1.2 (0.98–1.6)	
Median (IQR) peak serum C-reactive protein (mg/L)	25 (13–56)	
Radiological		
Chest X-ray performed	13 (41)	
Consolidation	8 (25)	104 (24)
Management		
Admission to level 3 care	1 (3.1)	41 (9.6)
Peak oxygen requirement		
None	27 (84)	
2 L nasal cannulae	2 (6.3)	
5 L face mask	1 (3.1)	
10 L non-rebreathe mask	1 (3.1)	
Non-invasive respiratory support	1 (3.1)	

UKOSS: UK obstetric surveillance system; PCR: polymerase chain reaction; IQR: interquartile range.

### Pregnancy outcomes

The maternal and fetal outcomes of women diagnosed with SARS-CoV-2 and our departmental comparator group are displayed in Table 3. Of 32 women, delivery data is available for 30; one women has yet to deliver at time of writing, and one woman delivered abroad. There was one maternal death during the study; this woman in early second trimester was self-isolating at home for presumed SARS-CoV-2 infection due to fever and cough before rapidly deteriorating at home. No post-mortem was performed on request of the family. There was a higher rate of delivery by emergency caesarean section compared with background departmental rates. Only one emergency caesarean delivery was performed due to clinical features of SARS-CoV-2 infection. Two elective caesarean procedures were delayed due to infection control concerns which resulted in emergency procedures.

**Table 3.** Maternal and neonatal outcomes of pregnant women diagnosed with SARS-CoV-2 and background departmental figures.

	Departmental ratesNumber (%)	SARS-CoV-2 infection Newham	Crude odds ratio (95% CI)	*p*-value
	*n* = 1268	*n* = 30		
Maternal				
Maternal death	0	1		
Mode of delivery				
SVD	745 (59)	14 (47)	0.61 (0.28–1.36)	0.18
Instrumental	159 (13)	4 (13)	1.07 (0.27–3.15)	0.90
All caesarean section	359 (28)	12 (40)	1.69 (0.73–3.74)	0.16
Elective caesarean	117 (9.2)	2 (6.7)	0.70 (0.08–2.85)	0.63
Emergency caesarean	242 (19)	10 (33)	2.12 (0.87–4.82)	0.05
Missing	5 (0.4)	–	–	–
Indication for caesarean due to SARS-CoV-2	–	1 (3.3)		
Neonatal				
Perinatal death	16 (1.3)	1 (0.03)	–	–
Stillbirth	10 (0.8)	0 (0.0)	–	–
Neonatal death	6 (0.5)	0 (0.0)	–	–
Gestational age, weeks^+ days^ (median, IQR)	39^+0^ (38–40)	38^+4^ (37–40)	–	–
Term	1168 (92)	24 (80)		
<37 weeks	117 (9.2)	6 (20)	2.46 (0.81–6.33)	0.05
<34 weeks	24 (1.9)	4 (13)	7.97 (1.87–25.6)	<0.001
Birthweight (mean ±SD)	3199 ±757	2940 ±668	–	0.06
Birthweight centile (mean ±SD)	56.1 ±28.2	49.6 ±33.2	–	0.22
Birthweight centile (median, IQR)	58.7, 33-80	61.5, 15-79	–	–
Low birth weight (<2500 g)	121 (9.5)	5 (17)	1.90 (0.56–5.16)	0.19
Apgar 5 min (Median, IQR)	10 (10–10)	10 (10–10)		
Neonatal admission	113 (8.9)	12 (40)	6.81 (2.91–15.3)	<0.001
Neonatal SARS-CoV-2 swab	–	7 (23)		
Positive		1		

There was one perinatal death, associated with the aforementioned maternal death. This is compared to a rate of 12.6/1000 births in the preceding three months. There was no significant difference in the median gestational age at delivery, birthweight centile or Apgar scores at 5 min of babies born to women with coronavirus disease compared to background departmental rates. However, a larger proportion of neonates were born preterm or very preterm, and a larger proportion admitted to the neonatal unit. Twelve neonates were admitted; three due to prematurity, five for treatment with intravenous antibiotics, two due to neonatal jaundice, one due to maternal condition, and another after 20 days due to poor feeding and weight loss.

Seven neonates underwent a SARS-CoV-2 nasopharyngeal swab, all due to neonatal fever. Of these, one swab was positive; this neonate was born at 35 weeks and 3 days of gestation to a mother with type 2 diabetes mellitus and BMI 40 kg/m^2^ and treated with insulin. The early neonatal period was complicated by hypoglycaemia and fever. The baby was admitted to the neonatal unit for intravenous antibiotics for presumed sepsis. There was no respiratory distress and antibiotics were stopped after negative blood cultures. The baby was discharged after three days in good health.

## Discussion

### Main findings

In Newham, we report an estimated incidence of SARS-CoV-2 infection in pregnancy more than ten times the national average. In this area of high ethnic diversity and diabetes prevalence, a disproportionate number of women from Black and Asian backgrounds were diagnosed. However, there was no association with obesity or metabolic disorders in pregnancy. The clinical features and severity of illness are similar to the national surveillance data. Babies born to women diagnosed with coronavirus were more likely to be born preterm, by emergency caesarean section, or admitted to the neonatal unit compared to the background population.

### Strengths and limitations

This is the first study to directly compare pregnancy outcomes with an appropriate local comparator group. We provide a unique insight into associations within an area with high ethnic diversity, underlying health conditions and deprivation, which adds valuable detail to the national surveillance data. The study is limited by the sample size and small study effects should be considered. Some, but not all, of the pregnancy outcomes have been previously reported in the UKOSS report.^5^

### Interpretation in light of other evidence

We found that at the first peak of the UK outbreak, pregnant women in Newham were 10 times more likely to be diagnosed with coronavirus disease than the national average. This exemplifies the heterogeneity of transmission rates across the country. We found that pregnant women from Black and Asian backgrounds were disproportionately represented in our sample. The UKOSS study showed a similar over-representation of these groups, however they performed only a partial adjustment for location with a sensitivity analysis excluding some major conurbations where rates of coronavirus transmission have been higher.^5^ However, this does not take account of other areas where more severe outbreaks may occur in areas where ethnic diversity is higher. For example, they did not exclude conurbations in the North-East of England which had the highest age-standardised diagnosis rates in females in the country according to the Public Health England Report.^10^ Our study helps to resolve this limitation by showing that, even within an area of high ethnic diversity and deprivation, there remains an over-representation of women from Black and Asian ethnic groups. These associations within ethnically diverse areas were also found in a recent study assessing associations with death due SARS-CoV-2 in five hospitals within our area of East London (pre-print only).^16^ We note that all studies assessing ethnicity do so in the confines of the labels available. In our study, the five women coded as ‘White’ were all from Eastern Europe, all born outside of the UK, with two needing a language advocate. This reminds us that labels of ethnicity fall short in reflecting an individual’s socio-economic background.

Our study shows no association between hospital admission with SARS-CoV-2 and obesity and diabetes, which differs to the findings of the UKOSS study. The associations described in the national survey may be due to the high prevalence of metabolic disorders in geographical areas most affected, such as our own. Other studies have also described associations with obesity and diabetes, such as a large cohort of pregnant women in France^17^ and the recent OpenSAFELY study.^9^ However, these associations were with critical care admission in pregnant women and death in the general adult population respectively, so a direct comparison cannot be made. Notably, the OpenSAFELY study made use of large population data and was able to provide robust adjustment for geographical area and background demographic data. Therefore, whilst our data do not show an association with admission, enough other evidence exists that appropriate caution should be taken when managing pregnant women with these co-morbidities.

We found that women diagnosed in a deprived and ethnically diverse area were no more severely affected by infection with SARS-CoV-2 than women nationally. They had similar symptoms and, reassuringly, we do not report high rates of oxygen requirement or critical care admission. We show that few had the classical finding of lymphopenia, and relatively mild increases in c-reactive protein, as reported elsewhere.

We found no difference in perinatal death or median gestational age between the two groups. This reflects the UKOSS data on perinatal death and gestational age. However, we found a positive trend towards preterm birth in babies born to women diagnosed with coronavirus disease compared to our own background departmental figures; however, this may be limited by numbers. The trend towards lower birthweight seems attributable to gestational age as birthweight centiles for gestational age are comparable. It is not known whether SARS-CoV-2 causes placental disease and intra-uterine growth restriction, as was considered the case with MERS; however, a recent report of isolation of the virus from the placenta of a preterm infant warrants further work.^3,18^ There is little other data available on birthweight, and the UKOSS study did not report this outcome, nor did a study of 657 pregnancies in France.^17^ A study of 116 pregnant women in Wuhan and also described reassuring pregnancy outcomes, including birthweight, however they did not provide a local comparator group.^6^ We report a higher rate of emergency caesarean delivery in our study which may be explained by delayed elective procedures due to infection control concerns. Only one women had an early pre-labour procedure due to coronavirus disease. The high rates of neonatal admission are seen in these other studies, and may reflect increased caution in the face of a novel disease.

### Summary

Pregnant women from Black and Asian backgrounds are more at risk of admission with coronavirus disease, even within an area of high ethnic diversity. Reassuringly, women are not more severely affected than women nationally. Whilst many pregnancy outcomes are similar to background departmental rates, the association with preterm birth warrants further work. The UK is currently experiencing further waves of SARS-CoV-2 infection in the absence of widespread vaccination.We now have overwhelming evidence that not only do disparities based on ethnicity and deprivation exist, but that they matter. We are aware of which pregnant women are most at risk, and should prepare our services accordingly.

## References

[1] The ANZIC Influenza Investigators and Australasian Maternity Outcomes Surveillance System. Critical illness due to 2009 A/H1N1 influenza in pregnant and postpartum women: population based cohort study. BMJ 340: c1279–c1279. 10.1136/bmj.c1279 PMC2841744 20299694

[2] Siston AM. Pandemic 2009 influenza A (H1N1) virus illness among pregnant women in the United States. JAMA 2010; 303: 1517. 20407061 10.1001/jama.2010.479 PMC5823273

[3] Wong SF Chow KM Leung TN , et al. Pregnancy and perinatal outcomes of women with severe acute respiratory syndrome. Am J Obstet Gynecol 2004; 191: 292–297. 15295381 10.1016/j.ajog.2003.11.019 PMC7137614

[4] Alfaraj SH Al-Tawfiq JA Memish ZA. Middle East Respiratory Syndrome Coronavirus (MERS-CoV) infection during pregnancy: report of two cases & review of the literature. J Microbiol Immunol Infect 2020; 52: 501–503. 10.1016/j.jmii.2018.04.005 PMC7128238 29907538

[5] Knight M Bunch K Vousden N , et al. Characteristics and outcomes of pregnant women admitted to hospital with confirmed SARS-CoV-2 infection in UK: national population based cohort study. BMJ 2020. DOI: 10.1101/2020.05.08.20089268. 10.1136/bmj.m2107 PMC7277610 32513659

[6] Yan J Guo J Fan C , et al. Coronavirus disease 2019 (COVID-19) in pregnant women: a report based on 116 cases. Am J Obstet Gynecol 2020. 10.1016/j.ajog.2020.04.014 PMC7177142 32335053

[7] Intensive Care and National Audit & Research Centre (ICNARC) Report, www.icnarc.org/Our-Audit/Audits/Cmp/Reports

[8] Aldridge RW Lewer D Katikireddi SV , et al. Black, Asian and Minority Ethnic groups in England are at increased risk of death from COVID-19: indirect standardisation of NHS mortality data. Wellcome Open Res 2020; 5: 88. 32613083 10.12688/wellcomeopenres.15922.1 PMC7317462

[9] Collaborative TO Williamson E Walker AJ , et al. OpenSAFELY: factors associated with COVID-19-related hospital death in the linked electronic health records of 17 million adult NHS patients. Epidemiology 2020.

[10] PHE. Disparities in the risk and outcomes of COVID-19; Public Health England, https://assets.publishing.service.gov.uk/government/uploads/system/uploads/attachment_data/file/891116/disparities_review.pdf

[11] Office for National Statistics (ONS), www.ons.gov.uk/peoplepopulationandcommunity/birthsdeathsandmarriages/deaths/bulletins/deathsinvolvingcovid19bylocalareasanddeprivation/deathsoccurringbetween1marchand17april?hootPostID=f8f83cc51cba7b7e20edce0e1993cadf

[12] Newham Government Website, www.newham.info/population/

[13] Ethnicity Facts and Figures Government Website, www.ethnicity-facts-figures.service.gov.uk/uk-population-by-ethnicity

[14] National Diabetes Audit (NDA); January to December 2019, https://digital.nhs.uk/data-and-information/publications/statistical/national-diabetes-audit/care-processes-and-treatment-targets-january-to-september-2019

[15] Intergrowth 21 – Standards and tools, https://intergrowth21.tghn.org/standards-tools/ (Accessed 22nd October 2020).

[16] Apea VJ Wan YI Dhairyawan R , et al. Ethnicity and outcomes in patients hospitalised with COVID-19 infection in East London: an observational cohort study. Epidemiology 2020. 10.1136/bmjopen-2020-042140 PMC7813387 33455936

[17] Kayem G Alessandrini V Azria E , et al. A snapshot of the Covid-19 pandemic among pregnant women in France. J Gynecol Obstet Hum Reprod 2020; 4: 101826. Jun 10.1016/j.jogoh.2020.101826 PMC7270811 32505805

[18] Baud D Greub G Favre G , et al. Second-trimester miscarriage in a pregnant woman with SARS-CoV-2 infection. JAMA 2020; 16. 10.1001/jama.2020.7233 PMC7193526 32352491

